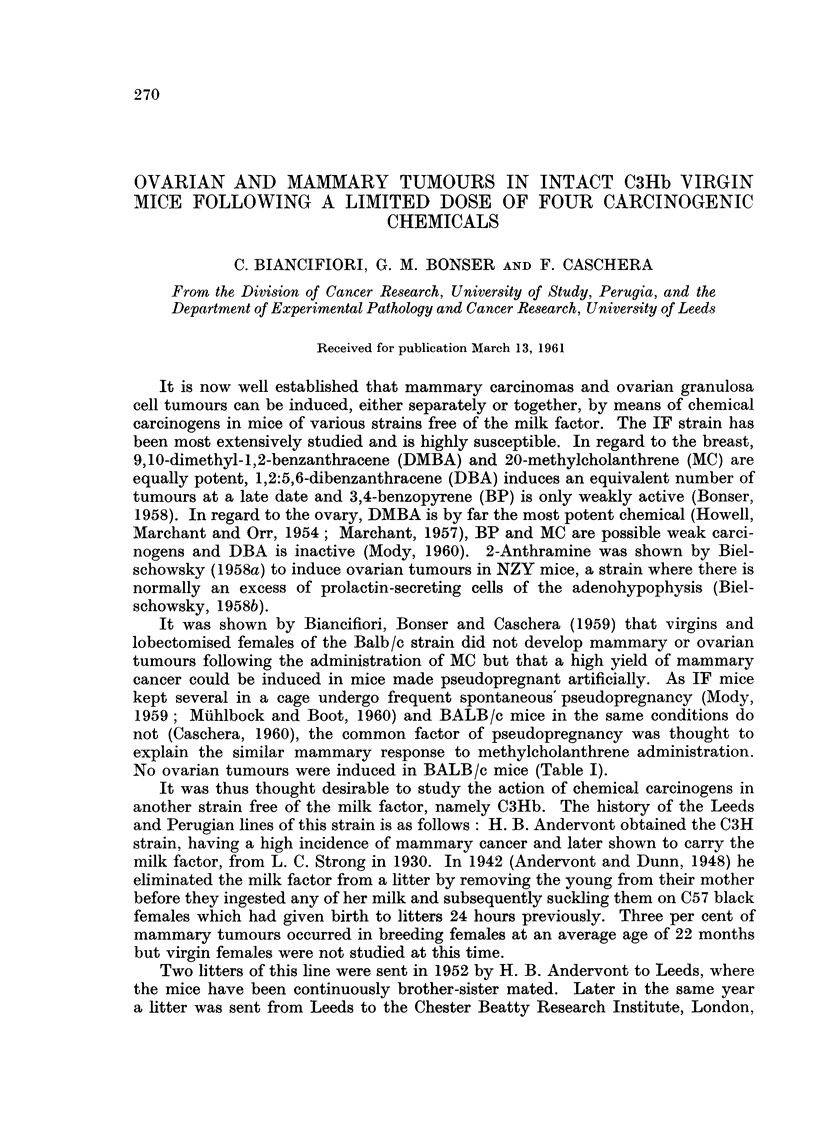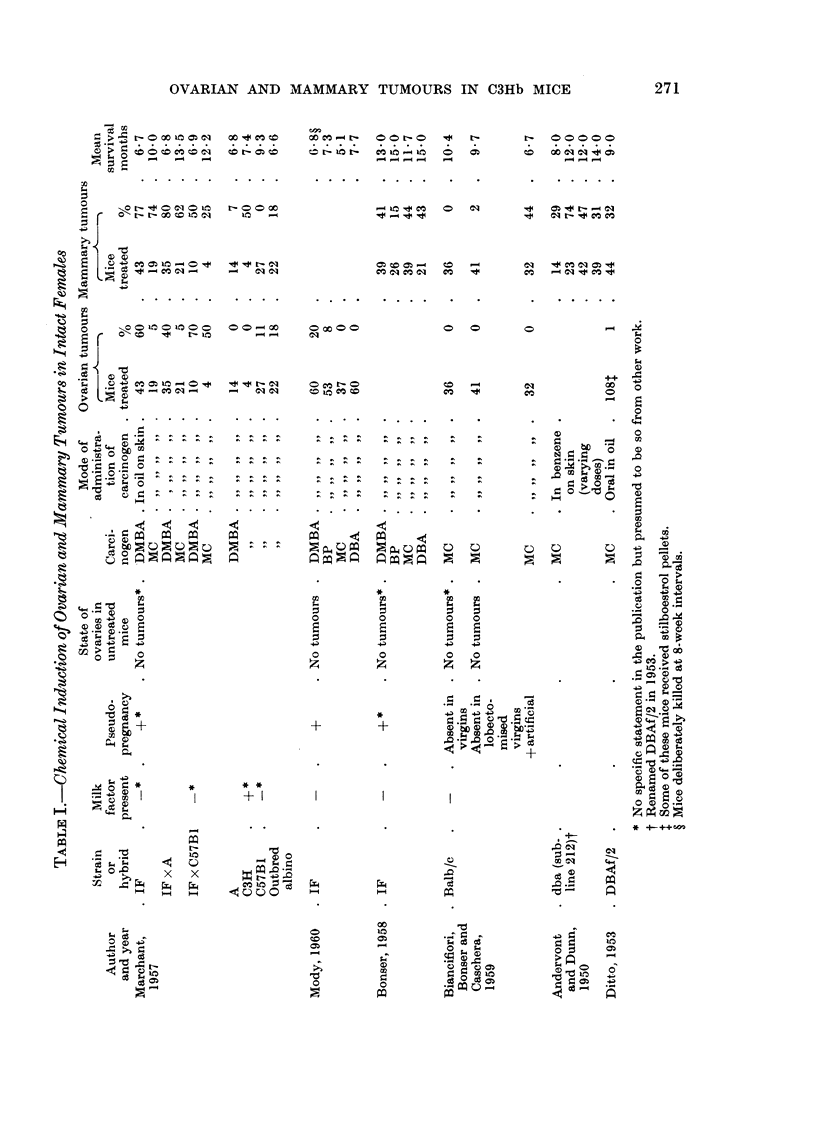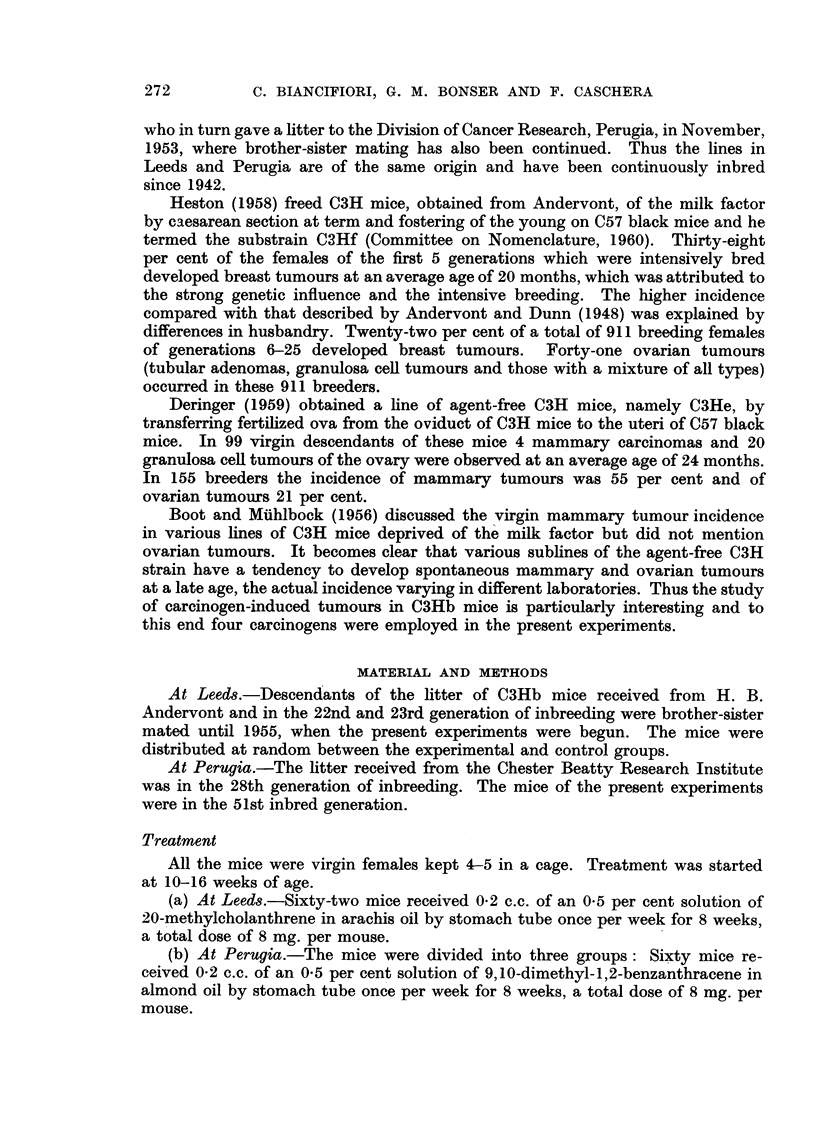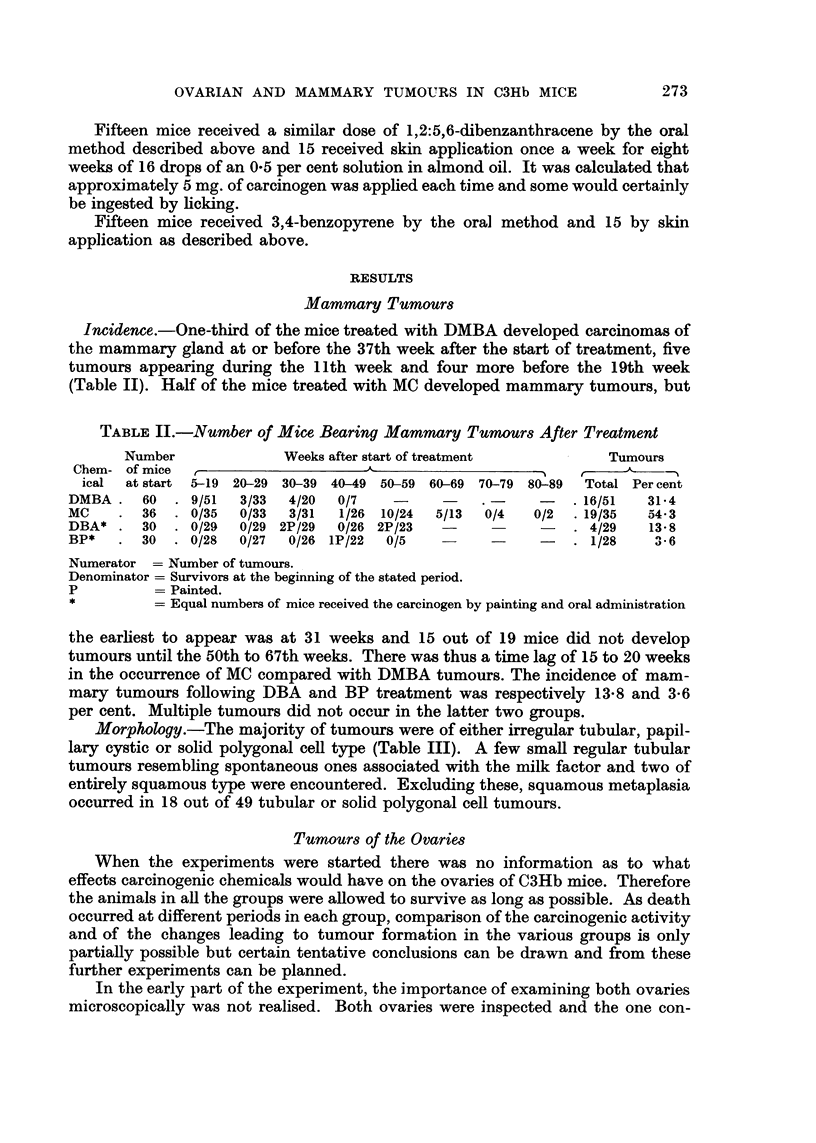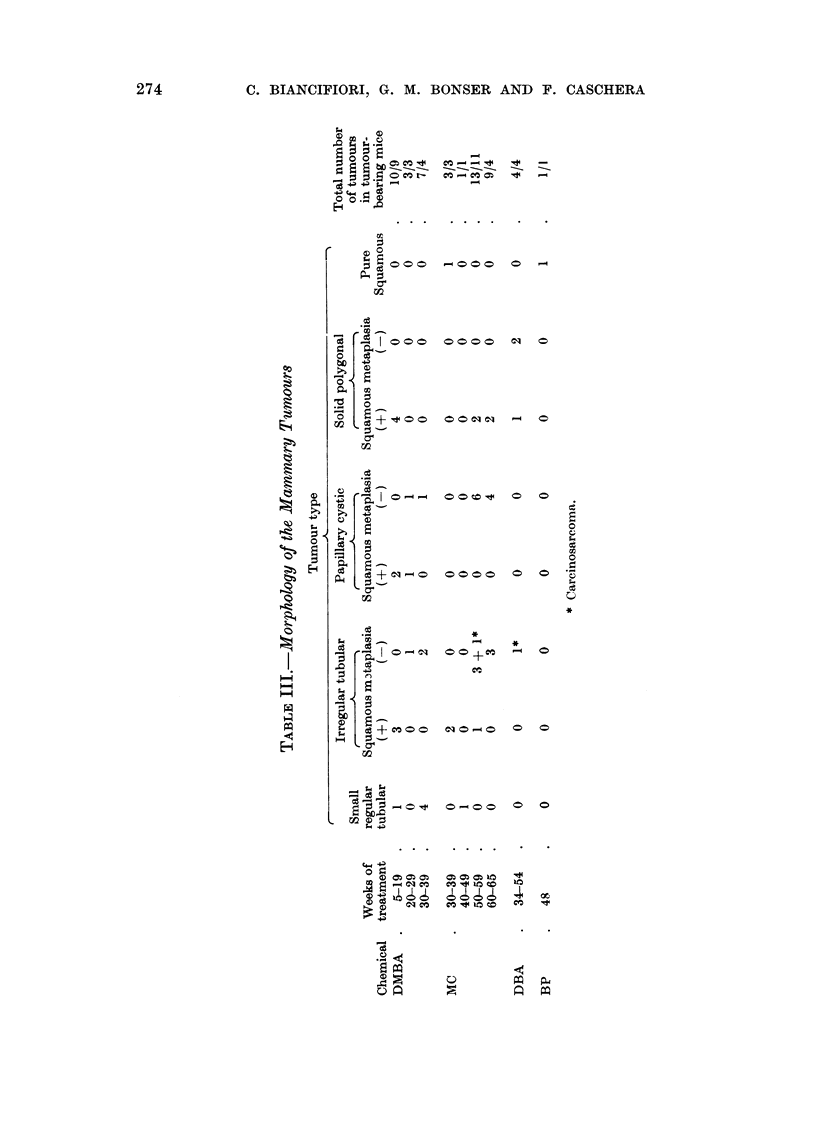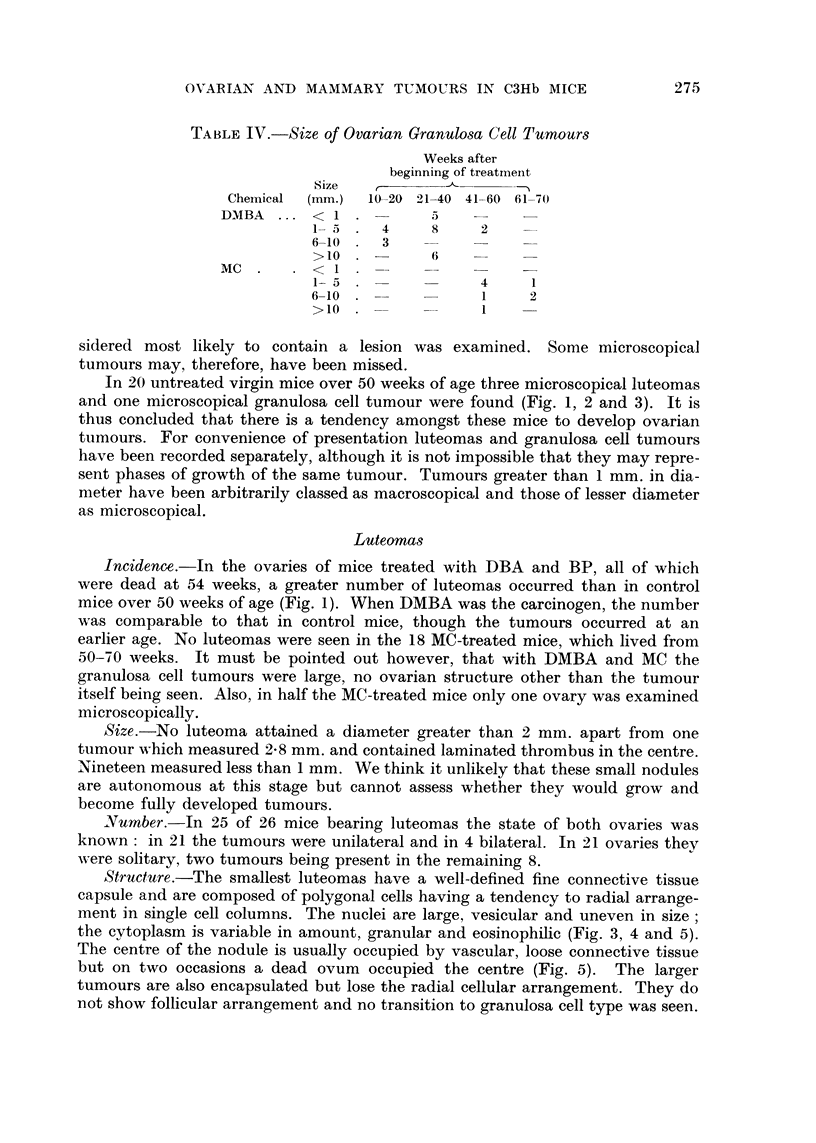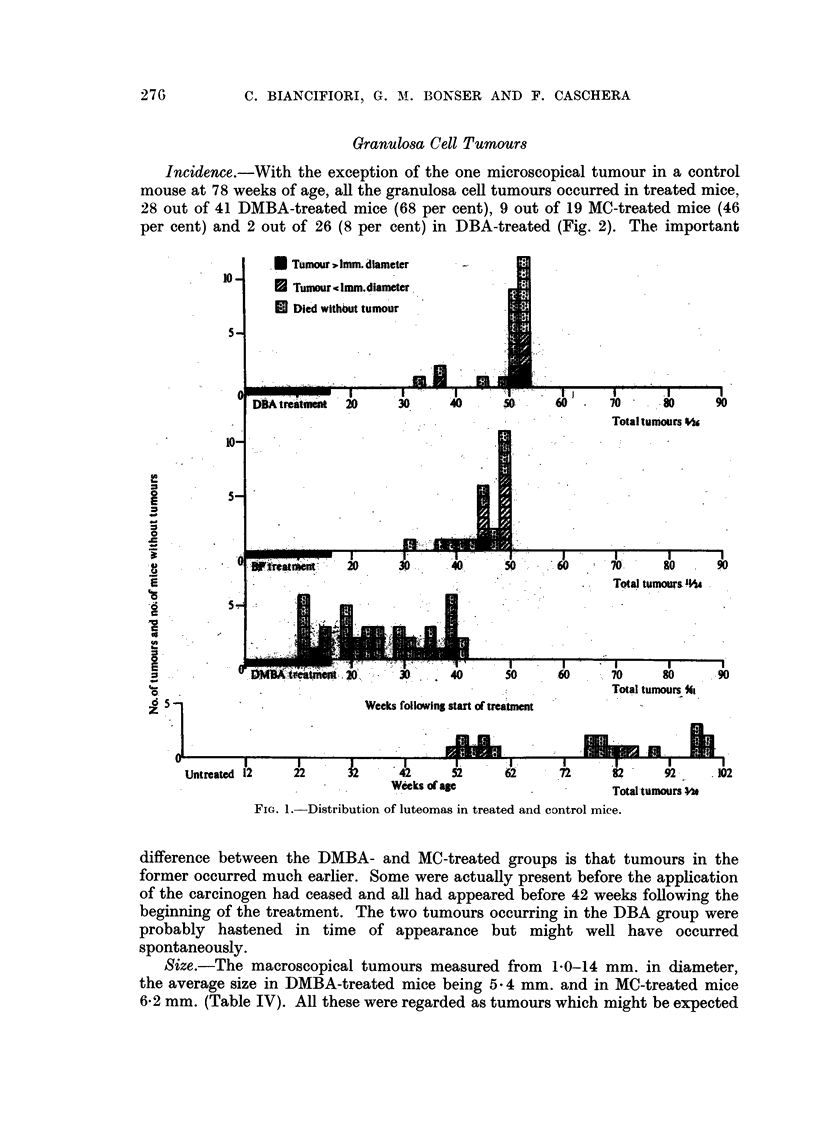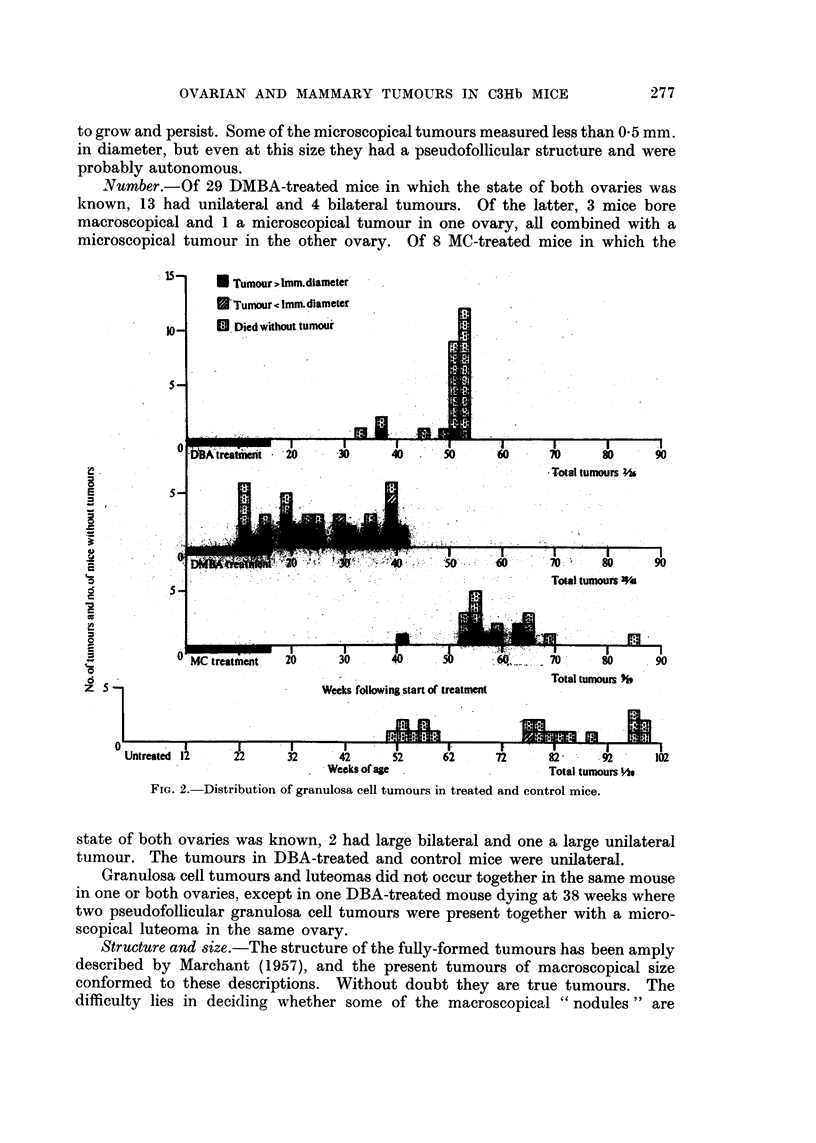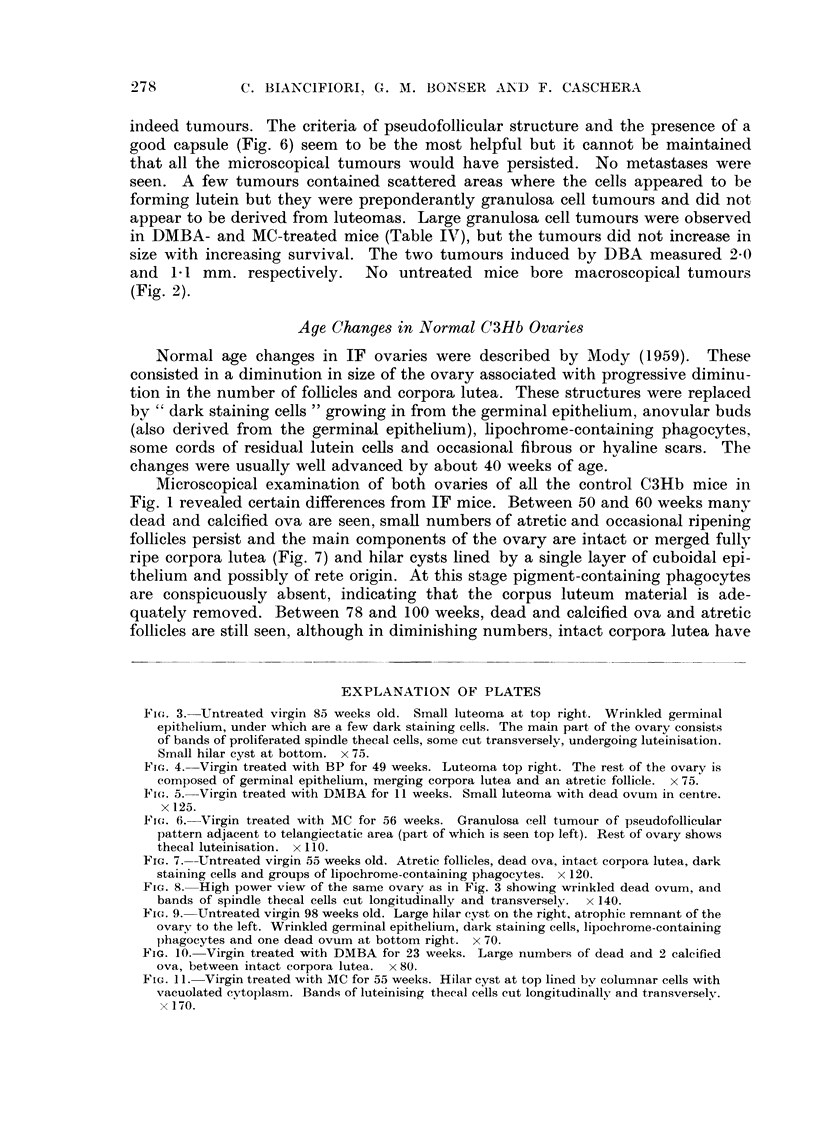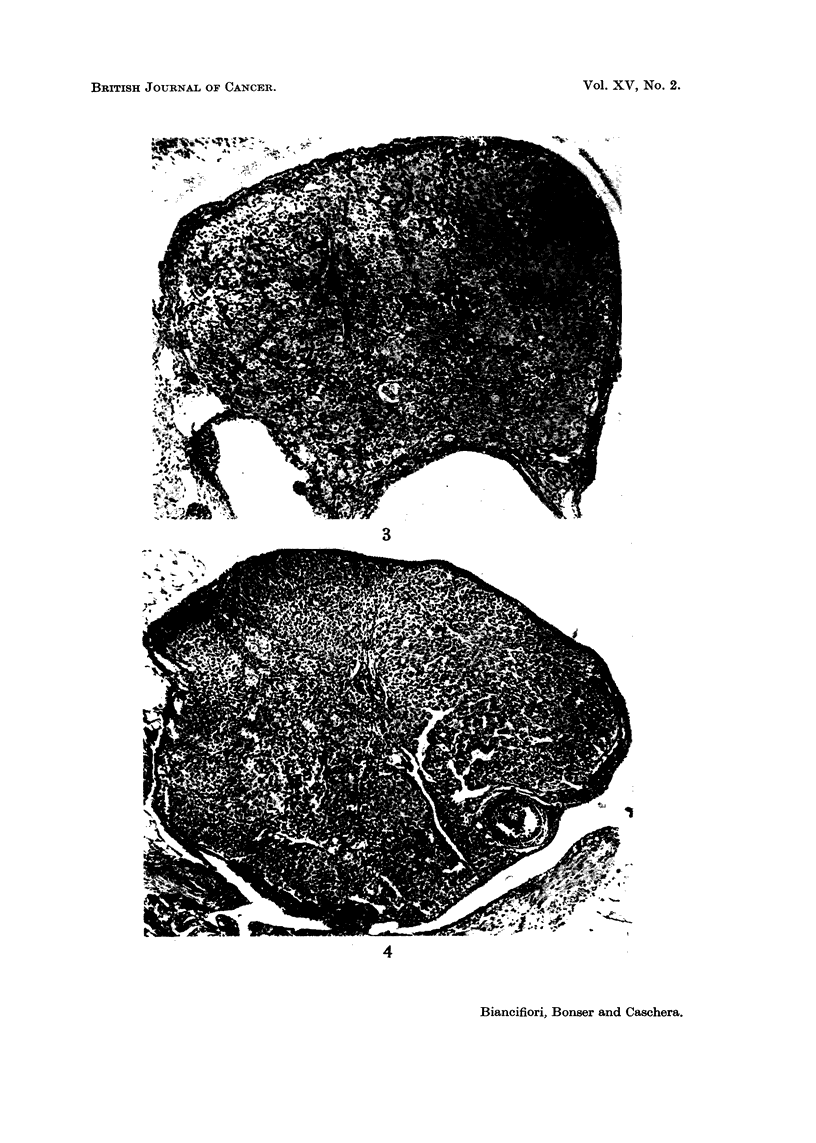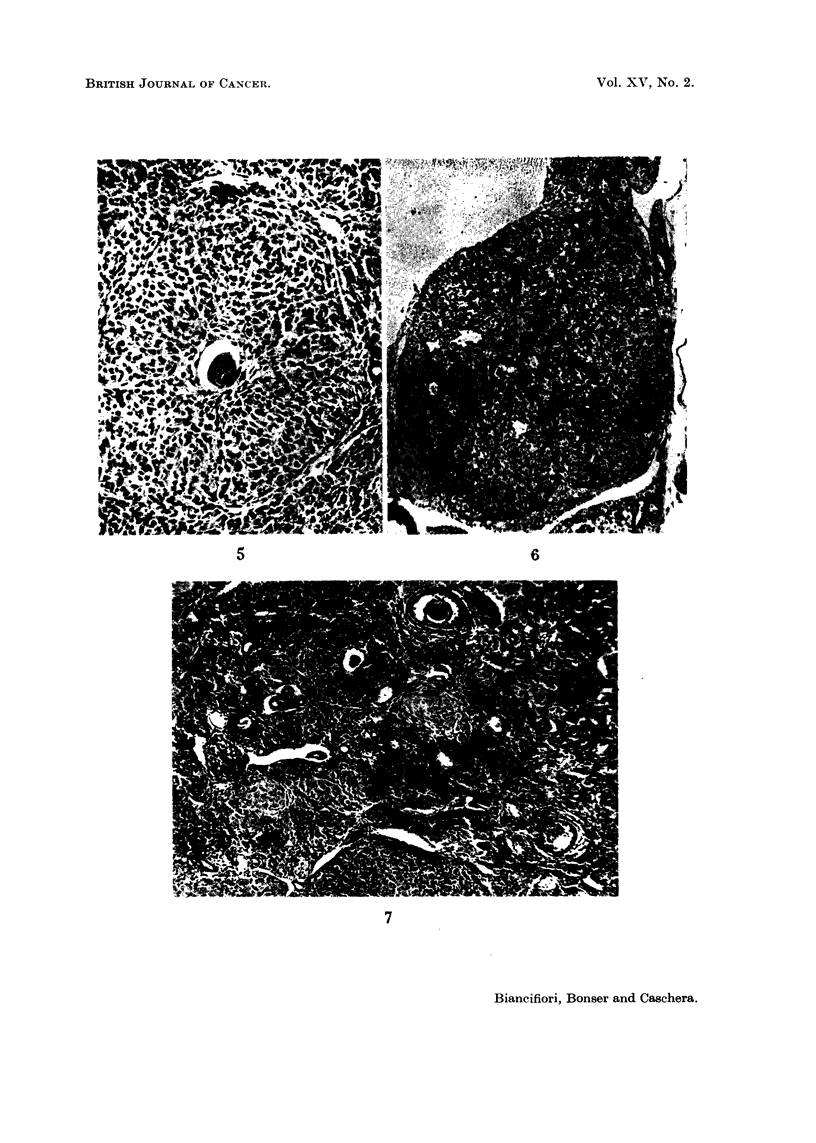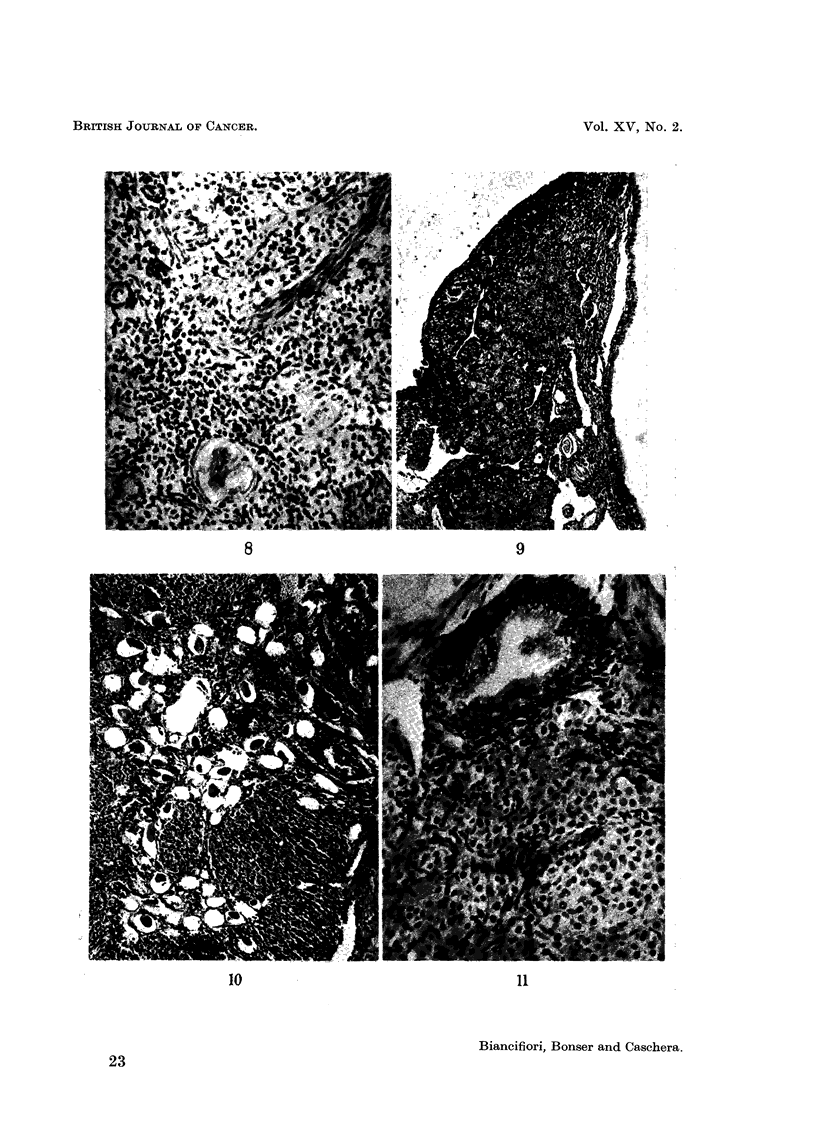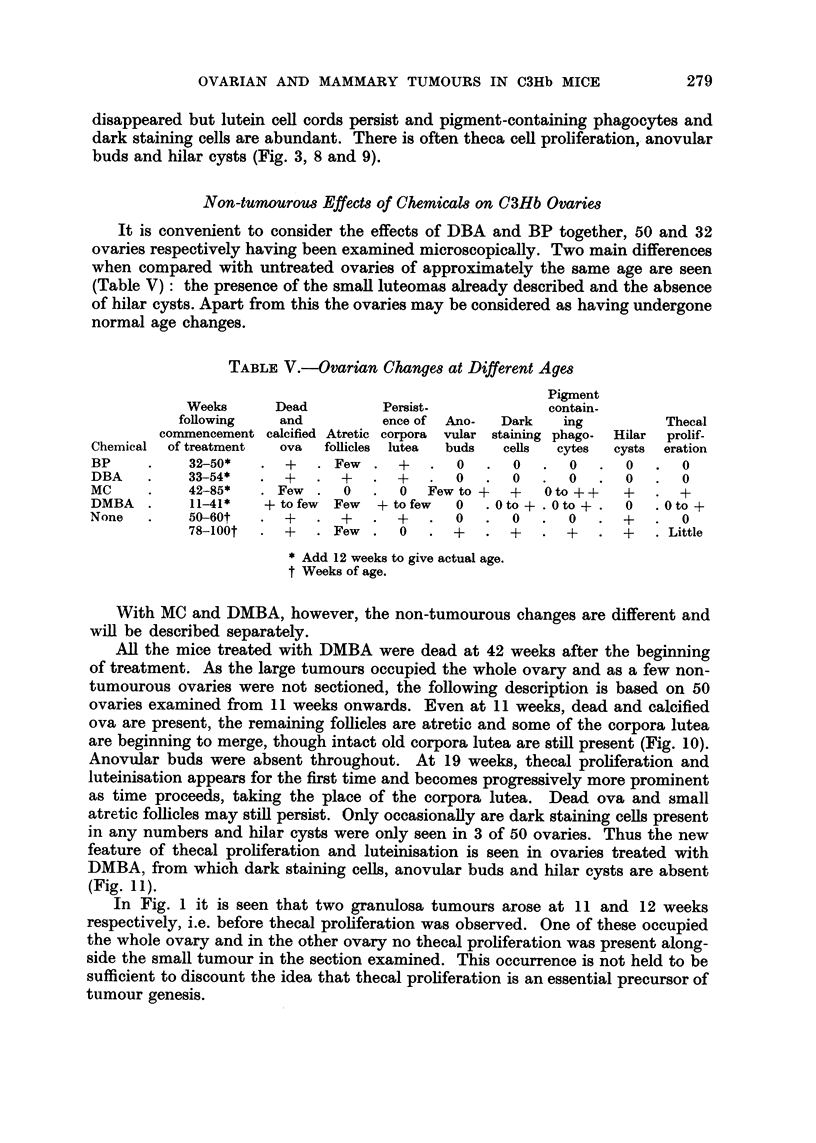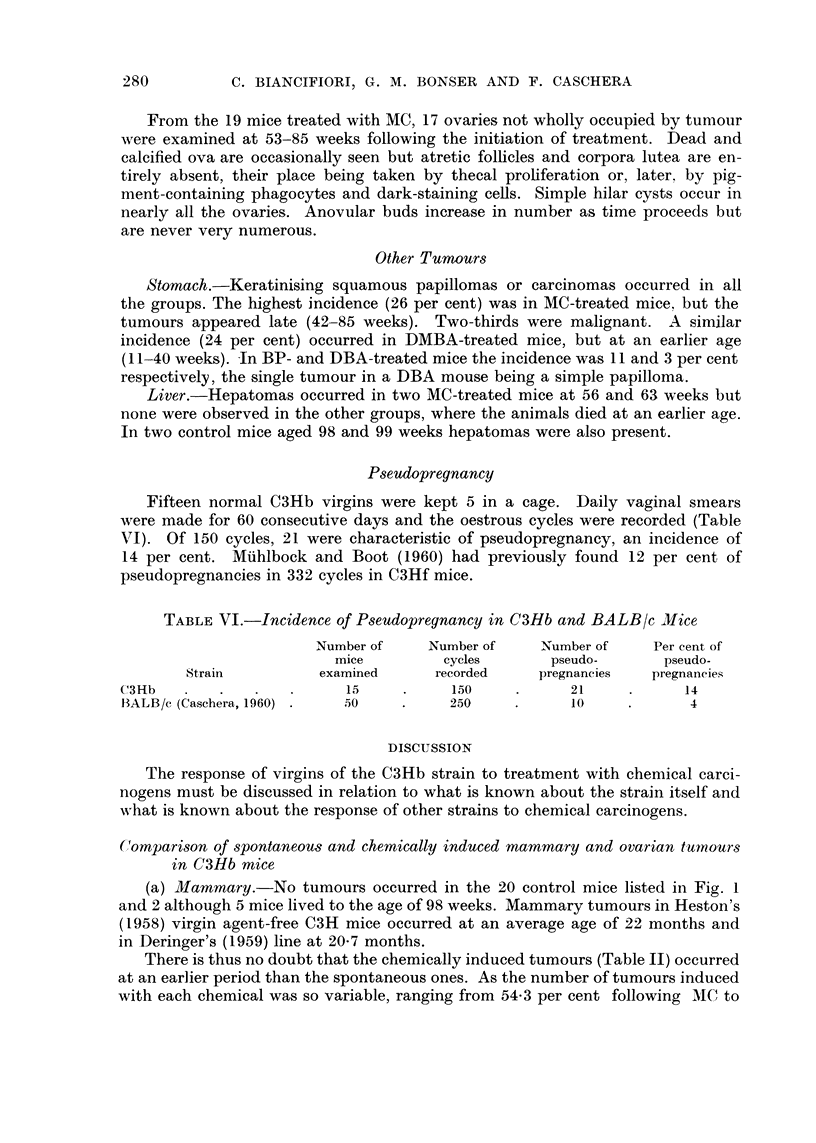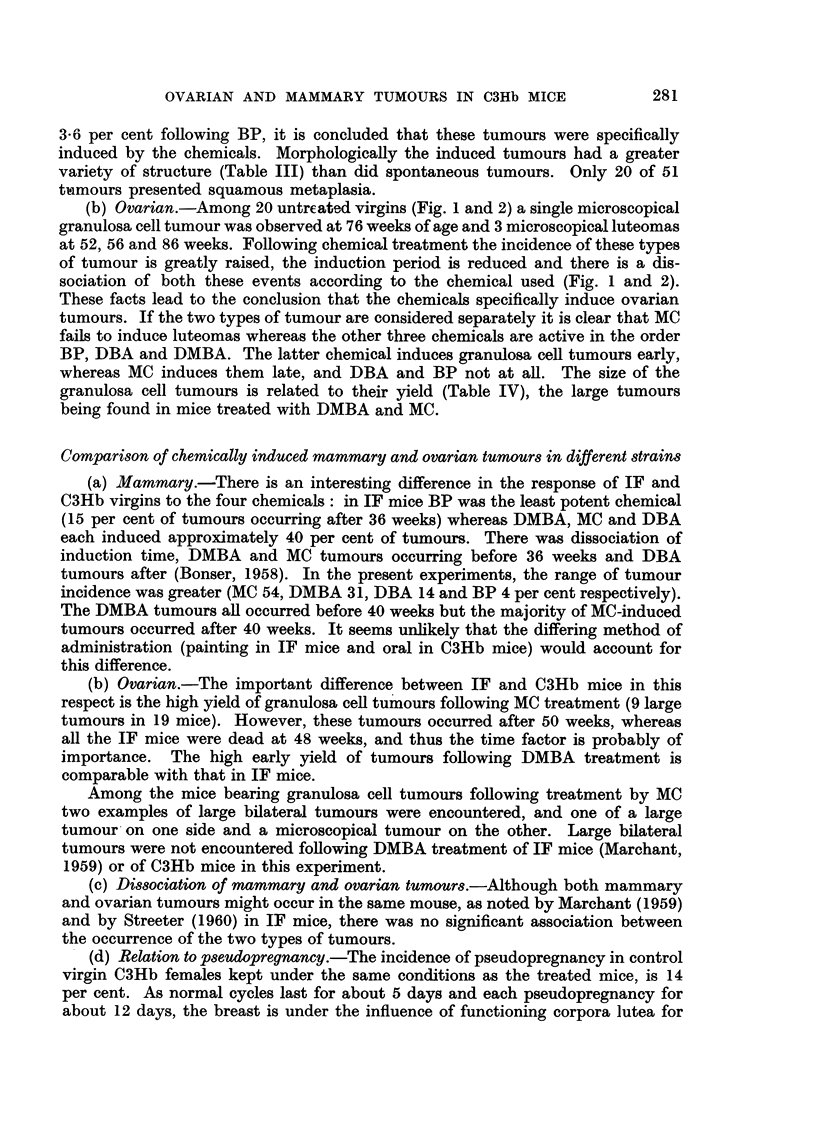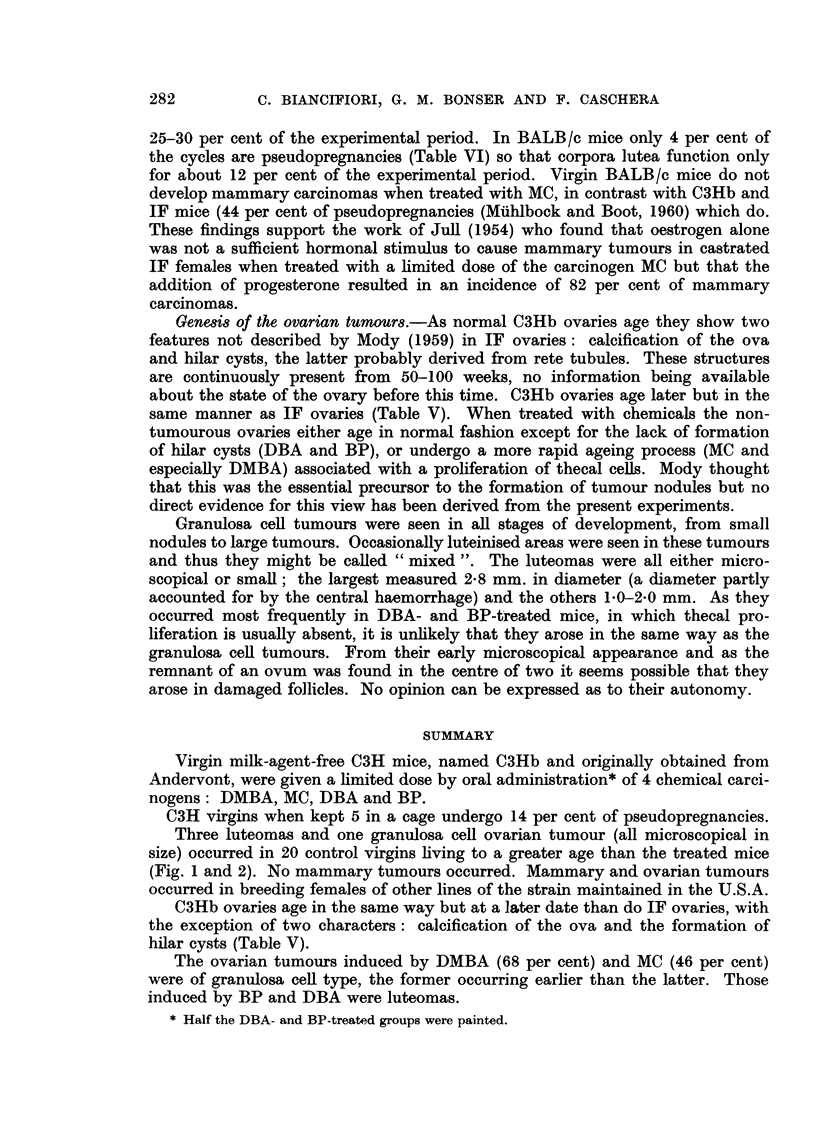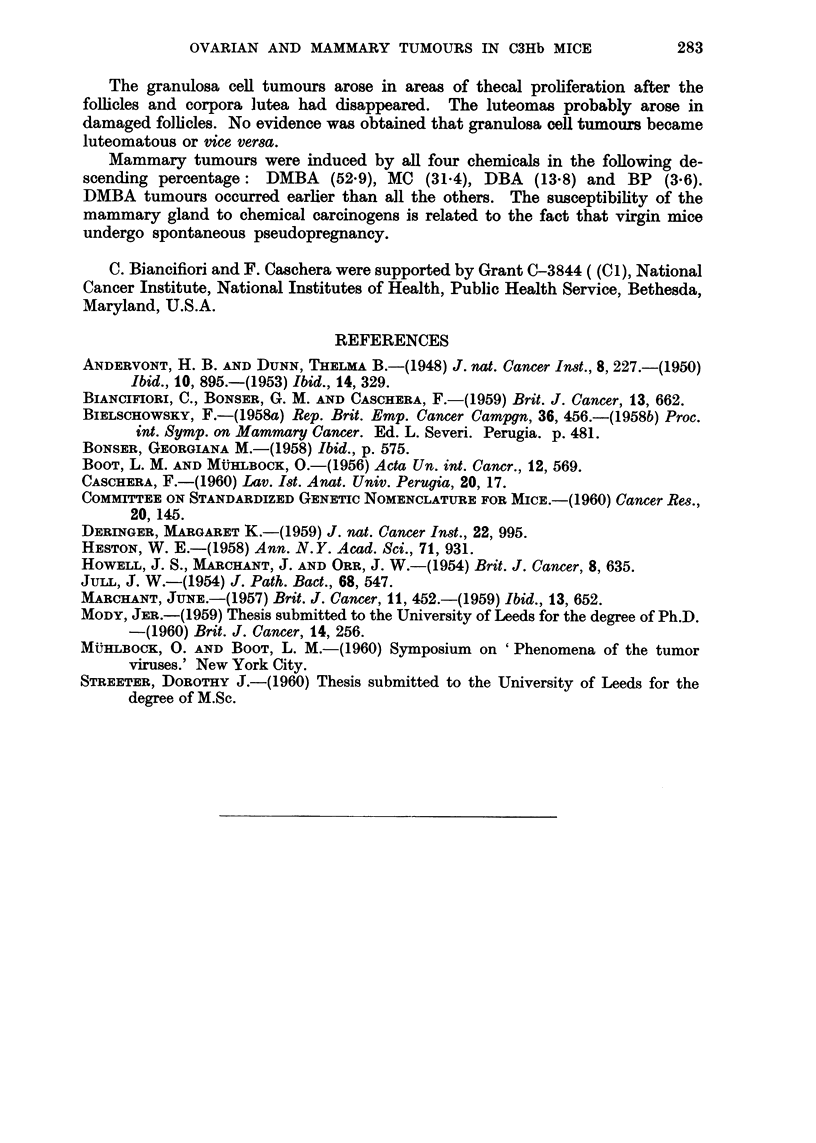# Ovarian and Mammary Tumours in Intact C3Hb Virgin Mice following a Limited Dose of Four Carcinogenic Chemicals

**DOI:** 10.1038/bjc.1961.34

**Published:** 1961-06

**Authors:** C. Biancifiori, G. M. Bonser, F. Caschera

## Abstract

**Images:**


### OVARIAN AND MAMMARY TUMOURS IN INTACT C3Hb VIRGIN MICE FOLLOWING A LIMITED DOSE OF FOUR CARCINOGENIC CHEMICALS

C. BIANCIFIORI, G. M. BONSER AND F. CASCHERA

*From the Division of Cancer Research, University of Study, Perugia, and the Department of Experimental Pathology and Cancer Research, University of Leeds*

Received for publication March 13, 1961

It is now well established that mammary carcinomas and ovarian granulosa cell tumours can be induced, either separately or together, by means of chemical carcinogens in mice of various strains free of the milk factor. The IF strain has been most extensively studied and is highly susceptible. In regard to the breast, 9,10-dimethyl-1,2-benzanthracene (DMBA) and 20-methylcholanthrene (MC) are equally potent, 1,2:5,6-dibenzanthracene (DBA) induces an equivalent number of tumours at a late date and 3,4-benzopyrene (BP) is only weakly active (Bonser, 1958). In regard to the ovary, DMBA is by far the most potent chemical (Howell, Marchant and Orr, 1954 ; Marchant, 1957), BP and MC are possible weak carcinogens and DBA is inactive (Mody, 1960). 2-Anthramine was shown by Bielschowsky (1958*a*) to induce ovarian tumours in NZY mice, a strain where there is normally an excess of prolactin-secreting cells of the adenohypophysis (Bielschowsky, 1958*b*).

It was shown by Biancifiori, Bonser and Caschera (1959) that virgins and lobectomised females of the Balb/c strain did not develop mammary or ovarian tumours following the administration of MC but that a high yield of mammary cancer could be induced in mice made pseudopregnant artificially. As IF mice kept several in a cage undergo frequent spontaneous pseudopregnancy (Mody, 1959 ; Mühlbock and Boot, 1960) and BALB/c mice in the same conditions do not (Caschera, 1960), the common factor of pseudopregnancy was thought to explain the similar mammary response to methylcholanthrene administration. No ovarian tumours were induced in BALB/c mice (Table I).

It was thus thought desirable to study the action of chemical carcinogens in another strain free of the milk factor, namely C3Hb. The history of the Leeds and Perugian lines of this strain is as follows : H. B. Andervont obtained the C3H strain, having a high incidence of mammary cancer and later shown to carry the milk factor, from L. C. Strong in 1930. In 1942 (Andervont and Dunn, 1948) he eliminated the milk factor from a litter by removing the young from their mother before they ingested any of her milk and subsequently suckling them on C57 black females which had given birth to litters 24 hours previously. Three per cent of mammary tumours occurred in breeding females at an average age of 22 months but virgin females were not studied at this time.

Two litters of this line were sent in 1952 by H. B. Andervont to Leeds, where the mice have been continuously brother-sister mated. Later in the same year a litter was sent from Leeds to the Chester Beatty Research Institute, London,

TABLE I.—*Chemical Induction of Ovarian and Mammary Tumours in Intact Females*

| Author and year | Strain or hybrid | Milk factor present | Pseudo-pregnancy | State of ovaries in untreated mice | Carcinogen | Mode of administration of carcinogen | Ovarian tumours: Mice treated | Ovarian tumours: % | Mammary tumours: Mice treated | Mammary tumours: % | Mean survival months |
|---|---|---|---|---|---|---|---|---|---|---|---|
| Marchant, 1957 | IF | –* | +* | No tumours* | DMBA | In oil on skin | 43 | 60 | 43 | 77 | 6·7 |
| | | | | | MC | ,, ,, ,, ,, | 19 | 5 | 19 | 74 | 10·0 |
| | IF × A | | | | DMBA | , ,, ,, ,, | 35 | 40 | 35 | 80 | 6·8 |
| | | | | | MC | ,, ,, ,, ,, | 21 | 5 | 21 | 62 | 13·5 |
| | IF × C57Bl | –* | | | DMBA | ,, ,, ,, ,, | 10 | 70 | 10 | 50 | 6·9 |
| | | | | | MC | ,, ,, ,, ,, | 4 | 50 | 4 | 25 | 12·2 |
| | A | | | | DMBA | ,, ,, ,, ,, | 14 | 0 | 14 | 7 | 6·8 |
| | C3H | +* | | | ,, | ,, ,, ,, ,, | 4 | 0 | 4 | 50 | 7·4 |
| | C57Bl | –* | | | ,, | ,, ,, ,, ,, | 27 | 11 | 27 | 0 | 9·3 |
| | Outbred albino | | | | ,, | ,, ,, ,, ,, | 22 | 18 | 22 | 18 | 6·6 |
| Mody, 1960 | IF | – | + | No tumours | DMBA | ,, ,, ,, ,, | 60 | 20 | | | 6·8§ |
| | | | | | BP | ,, ,, ,, ,, | 53 | 8 | | | 7·3 |
| | | | | | MC | ,, ,, ,, ,, | 37 | 0 | | | 5·1 |
| | | | | | DBA | ,, ,, ,, ,, | 60 | 0 | | | 7·7 |
| Bonser, 1958 | IF | – | +* | No tumours* | DMBA | ,, ,, ,, ,, | | | 39 | 41 | 13·0 |
| | | | | | BP | ,, ,, ,, ,, | | | 26 | 15 | 15·0 |
| | | | | | MC | ,, ,, ,, ,, | | | 39 | 44 | 11·7 |
| | | | | | DBA | ,, ,, ,, ,, | | | 21 | 43 | 15·0 |
| Biancifiori, Bonser and Caschera, 1959 | Balb/c | – | Absent in virgins | No tumours* | MC | ,, ,, ,, ,, | 36 | 0 | 36 | 0 | 10·4 |
| | | | Absent in lobectomised virgins | No tumours | MC | ,, ,, ,, ,, | 41 | 0 | 41 | 2 | 9·7 |
| | | | +artificial | | MC | ,, ,, ,, ,, | 32 | 0 | 32 | 44 | 6·7 |
| Andervont and Dunn, 1950 | dba (sub-line 212)† | | | | MC | In benzene on skin (varying doses) | | | 14 | 29 | 8·0 |
| | | | | | | | | | 23 | 74 | 12·0 |
| | | | | | | | | | 42 | 47 | 12·0 |
| | | | | | | | | | 39 | 31 | 14·0 |
| Ditto, 1953 | DBAf/2 | | | | MC | Oral in oil | 108‡ | 1 | 44 | 32 | 9·0 |

* No specific statement in the publication but presumed to be so from other work.

† Renamed DBAf/2 in 1953.

‡ Some of these mice received stilboestrol pellets.

§ Mice deliberately killed at 8-week intervals.

who in turn gave a litter to the Division of Cancer Research, Perugia, in November, 1953, where brother-sister mating has also been continued. Thus the lines in Leeds and Perugia are of the same origin and have been continuously inbred since 1942.

Heston (1958) freed C3H mice, obtained from Andervont, of the milk factor by caesarean section at term and fostering of the young on C57 black mice and he termed the substrain C3Hf (Committee on Nomenclature, 1960). Thirty-eight per cent of the females of the first 5 generations which were intensively bred developed breast tumours at an average age of 20 months, which was attributed to the strong genetic influence and the intensive breeding. The higher incidence compared with that described by Andervont and Dunn (1948) was explained by differences in husbandry. Twenty-two per cent of a total of 911 breeding females of generations 6–25 developed breast tumours. Forty-one ovarian tumours (tubular adenomas, granulosa cell tumours and those with a mixture of all types) occurred in these 911 breeders.

Deringer (1959) obtained a line of agent-free C3H mice, namely C3He, by transferring fertilized ova from the oviduct of C3H mice to the uteri of C57 black mice. In 99 virgin descendants of these mice 4 mammary carcinomas and 20 granulosa cell tumours of the ovary were observed at an average age of 24 months. In 155 breeders the incidence of mammary tumours was 55 per cent and of ovarian tumours 21 per cent.

Boot and Mühlbock (1956) discussed the virgin mammary tumour incidence in various lines of C3H mice deprived of the milk factor but did not mention ovarian tumours. It becomes clear that various sublines of the agent-free C3H strain have a tendency to develop spontaneous mammary and ovarian tumours at a late age, the actual incidence varying in different laboratories. Thus the study of carcinogen-induced tumours in C3Hb mice is particularly interesting and to this end four carcinogens were employed in the present experiments.

### MATERIAL AND METHODS

*At Leeds.*—Descendants of the litter of C3Hb mice received from H. B. Andervont and in the 22nd and 23rd generation of inbreeding were brother-sister mated until 1955, when the present experiments were begun. The mice were distributed at random between the experimental and control groups.

*At Perugia.*—The litter received from the Chester Beatty Research Institute was in the 28th generation of inbreeding. The mice of the present experiments were in the 51st inbred generation.

### *Treatment*

All the mice were virgin females kept 4–5 in a cage. Treatment was started at 10–16 weeks of age.

(a) *At Leeds.*—Sixty-two mice received 0·2 c.c. of an 0·5 per cent solution of 20-methylcholanthrene in arachis oil by stomach tube once per week for 8 weeks, a total dose of 8 mg. per mouse.

(b) *At Perugia.*—The mice were divided into three groups: Sixty mice received 0·2 c.c. of an 0·5 per cent solution of 9,10-dimethyl-1,2-benzanthracene in almond oil by stomach tube once per week for 8 weeks, a total dose of 8 mg. per mouse.

Fifteen mice received a similar dose of 1,2:5,6-dibenzanthracene by the oral method described above and 15 received skin application once a week for eight weeks of 16 drops of an 0·5 per cent solution in almond oil. It was calculated that approximately 5 mg. of carcinogen was applied each time and some would certainly be ingested by licking.

Fifteen mice received 3,4-benzopyrene by the oral method and 15 by skin application as described above.

### RESULTS

### *Mammary Tumours*

*Incidence.*—One-third of the mice treated with DMBA developed carcinomas of the mammary gland at or before the 37th week after the start of treatment, five tumours appearing during the 11th week and four more before the 19th week (Table II). Half of the mice treated with MC developed mammary tumours, but

TABLE II.—*Number of Mice Bearing Mammary Tumours After Treatment*

| Chemical | Number of mice at start | Weeks after start of treatment 5–19 | 20–29 | 30–39 | 40–49 | 50–59 | 60–69 | 70–79 | 80–89 | Tumours Total | Per cent |
|---|---|---|---|---|---|---|---|---|---|---|---|
| DMBA | 60 | 9/51 | 3/33 | 4/20 | 0/7 | — | — | — | — | 16/51 | 31·4 |
| MC | 36 | 0/35 | 0/33 | 3/31 | 1/26 | 10/24 | 5/13 | 0/4 | 0/2 | 19/35 | 54·3 |
| DBA* | 30 | 0/29 | 0/29 | 2P/29 | 0/26 | 2P/23 | — | — | — | 4/29 | 13·8 |
| BP* | 30 | 0/28 | 0/27 | 0/26 | 1P/22 | 0/5 | — | — | — | 1/28 | 3·6 |

Numerator = Number of tumours.
Denominator = Survivors at the beginning of the stated period.
P = Painted.
* = Equal numbers of mice received the carcinogen by painting and oral administration the earliest to appear was at 31 weeks and 15 out of 19 mice did not develop tumours until the 50th to 67th weeks. There was thus a time lag of 15 to 20 weeks in the occurrence of MC compared with DMBA tumours. The incidence of mammary tumours following DBA and BP treatment was respectively 13·8 and 3·6 per cent. Multiple tumours did not occur in the latter two groups.

*Morphology.*—The majority of tumours were of either irregular tubular, papillary cystic or solid polygonal cell type (Table III). A few small regular tubular tumours resembling spontaneous ones associated with the milk factor and two of entirely squamous type were encountered. Excluding these, squamous metaplasia occurred in 18 out of 49 tubular or solid polygonal cell tumours.

### *Tumours of the Ovaries*

When the experiments were started there was no information as to what effects carcinogenic chemicals would have on the ovaries of C3Hb mice. Therefore the animals in all the groups were allowed to survive as long as possible. As death occurred at different periods in each group, comparison of the carcinogenic activity and of the changes leading to tumour formation in the various groups is only partially possible but certain tentative conclusions can be drawn and from these further experiments can be planned.

In the early part of the experiment, the importance of examining both ovaries microscopically was not realised. Both ovaries were inspected and the one con-

TABLE III.—*Morphology of the Mammary Tumours*

| Chemical | Weeks of treatment | Tumour type: Small regular tubular | Irregular tubular: Squamous metaplasia (+) | Irregular tubular: Squamous metaplasia (−) | Papillary cystic: Squamous metaplasia (+) | Papillary cystic: Squamous metaplasia (−) | Solid polygonal: Squamous metaplasia (+) | Solid polygonal: Squamous metaplasia (−) | Pure Squamous | Total number of tumours in tumour-bearing mice |
|---|---|---|---|---|---|---|---|---|---|---|
| DMBA | 5–19 | 1 | 3 | 0 | 2 | 0 | 4 | 0 | 0 | 10/9 |
| | 20–29 | 0 | 0 | 1 | 1 | 1 | 0 | 0 | 0 | 3/3 |
| | 30–39 | 4 | 0 | 2 | 0 | 1 | 0 | 0 | 0 | 7/4 |
| MC | 30–39 | 0 | 2 | 0 | 0 | 0 | 0 | 0 | 1 | 3/3 |
| | 40–49 | 1 | 0 | 0 | 0 | 0 | 0 | 0 | 0 | 1/1 |
| | 50–59 | 0 | 1 | 3 + 1* | 0 | 6 | 2 | 0 | 0 | 13/11 |
| | 60–65 | 0 | 0 | 3 | 0 | 4 | 2 | 0 | 0 | 9/4 |
| DBA | 34–54 | 0 | 0 | 1* | 0 | 0 | 1 | 2 | 0 | 4/4 |
| BP | 48 | 0 | 0 | 0 | 0 | 0 | 0 | 0 | 1 | 1/1 |

* Carcinosarcoma.

TABLE IV.—*Size of Ovarian Granulosa Cell Tumours*

| Chemical | Size (mm.) | Weeks after beginning of treatment 10–20 | 21–40 | 41–60 | 61–70 |
|---|---|---|---|---|---|
| DMBA | < 1 | — | 5 | — | — |
| | 1–5 | 4 | 8 | 2 | — |
| | 6–10 | 3 | — | — | — |
| | >10 | — | 6 | — | — |
| MC | < 1 | — | — | — | — |
| | 1–5 | — | — | 4 | 1 |
| | 6–10 | — | — | 1 | 2 |
| | >10 | — | — | 1 | — |

sidered most likely to contain a lesion was examined. Some microscopical tumours may, therefore, have been missed.

In 20 untreated virgin mice over 50 weeks of age three microscopical luteomas and one microscopical granulosa cell tumour were found (Fig. 1, 2 and 3). It is thus concluded that there is a tendency amongst these mice to develop ovarian tumours. For convenience of presentation luteomas and granulosa cell tumours have been recorded separately, although it is not impossible that they may represent phases of growth of the same tumour. Tumours greater than 1 mm. in diameter have been arbitrarily classed as macroscopical and those of lesser diameter as microscopical.

### *Luteomas*

*Incidence.*—In the ovaries of mice treated with DBA and BP, all of which were dead at 54 weeks, a greater number of luteomas occurred than in control mice over 50 weeks of age (Fig. 1). When DMBA was the carcinogen, the number was comparable to that in control mice, though the tumours occurred at an earlier age. No luteomas were seen in the 18 MC-treated mice, which lived from 50–70 weeks. It must be pointed out however, that with DMBA and MC the granulosa cell tumours were large, no ovarian structure other than the tumour itself being seen. Also, in half the MC-treated mice only one ovary was examined microscopically.

*Size.*—No luteoma attained a diameter greater than 2 mm. apart from one tumour which measured 2·8 mm. and contained laminated thrombus in the centre. Nineteen measured less than 1 mm. We think it unlikely that these small nodules are autonomous at this stage but cannot assess whether they would grow and become fully developed tumours.

*Number.*—In 25 of 26 mice bearing luteomas the state of both ovaries was known : in 21 the tumours were unilateral and in 4 bilateral. In 21 ovaries they were solitary, two tumours being present in the remaining 8.

*Structure.*—The smallest luteomas have a well-defined fine connective tissue capsule and are composed of polygonal cells having a tendency to radial arrangement in single cell columns. The nuclei are large, vesicular and uneven in size ; the cytoplasm is variable in amount, granular and eosinophilic (Fig. 3, 4 and 5). The centre of the nodule is usually occupied by vascular, loose connective tissue but on two occasions a dead ovum occupied the centre (Fig. 5). The larger tumours are also encapsulated but lose the radial cellular arrangement. They do not show follicular arrangement and no transition to granulosa cell type was seen.

### *Granulosa Cell Tumours*

*Incidence.*—With the exception of the one microscopical tumour in a control mouse at 78 weeks of age, all the granulosa cell tumours occurred in treated mice, 28 out of 41 DMBA-treated mice (68 per cent), 9 out of 19 MC-treated mice (46 per cent) and 2 out of 26 (8 per cent) in DBA-treated (Fig. 2). The important

FIG. 1.—Distribution of luteomas in treated and control mice.

difference between the DMBA- and MC-treated groups is that tumours in the former occurred much earlier. Some were actually present before the application of the carcinogen had ceased and all had appeared before 42 weeks following the beginning of the treatment. The two tumours occurring in the DBA group were probably hastened in time of appearance but might well have occurred spontaneously.

*Size.*—The macroscopical tumours measured from 1·0–14 mm. in diameter, the average size in DMBA-treated mice being 5·4 mm. and in MC-treated mice 6·2 mm. (Table IV). All these were regarded as tumours which might be expected to grow and persist. Some of the microscopical tumours measured less than 0·5 mm. in diameter, but even at this size they had a pseudofollicular structure and were probably autonomous.

*Number.*—Of 29 DMBA-treated mice in which the state of both ovaries was known, 13 had unilateral and 4 bilateral tumours. Of the latter, 3 mice bore macroscopical and 1 a microscopical tumour in one ovary, all combined with a microscopical tumour in the other ovary. Of 8 MC-treated mice in which the

FIG. 2.—Distribution of granulosa cell tumours in treated and control mice.

state of both ovaries was known, 2 had large bilateral and one a large unilateral tumour. The tumours in DBA-treated and control mice were unilateral.

Granulosa cell tumours and luteomas did not occur together in the same mouse in one or both ovaries, except in one DBA-treated mouse dying at 38 weeks where two pseudofollicular granulosa cell tumours were present together with a microscopical luteoma in the same ovary.

*Structure and size.*—The structure of the fully-formed tumours has been amply described by Marchant (1957), and the present tumours of macroscopical size conformed to these descriptions. Without doubt they are true tumours. The difficulty lies in deciding whether some of the macroscopical "nodules" are indeed tumours. The criteria of pseudofollicular structure and the presence of a good capsule (Fig. 6) seem to be the most helpful but it cannot be maintained that all the microscopical tumours would have persisted. No metastases were seen. A few tumours contained scattered areas where the cells appeared to be forming lutein but they were preponderantly granulosa cell tumours and did not appear to be derived from luteomas. Large granulosa cell tumours were observed in DMBA- and MC-treated mice (Table IV), but the tumours did not increase in size with increasing survival. The two tumours induced by DBA measured 2·0 and 1·1 mm. respectively. No untreated mice bore macroscopical tumours (Fig. 2).

### *Age Changes in Normal C3Hb Ovaries*

Normal age changes in IF ovaries were described by Mody (1959). These consisted in a diminution in size of the ovary associated with progressive diminution in the number of follicles and corpora lutea. These structures were replaced by " dark staining cells " growing in from the germinal epithelium, anovular buds (also derived from the germinal epithelium), lipochrome-containing phagocytes, some cords of residual lutein cells and occasional fibrous or hyaline scars. The changes were usually well advanced by about 40 weeks of age.

Microscopical examination of both ovaries of all the control C3Hb mice in Fig. 1 revealed certain differences from IF mice. Between 50 and 60 weeks many dead and calcified ova are seen, small numbers of atretic and occasional ripening follicles persist and the main components of the ovary are intact or merged fully ripe corpora lutea (Fig. 7) and hilar cysts lined by a single layer of cuboidal epithelium and possibly of rete origin. At this stage pigment-containing phagocytes are conspicuously absent, indicating that the corpus luteum material is adequately removed. Between 78 and 100 weeks, dead and calcified ova and atretic follicles are still seen, although in diminishing numbers, intact corpora lutea have

---

EXPLANATION OF PLATES

FIG. 3.—Untreated virgin 85 weeks old. Small luteoma at top right. Wrinkled germinal epithelium, under which are a few dark staining cells. The main part of the ovary consists of bands of proliferated spindle thecal cells, some cut transversely, undergoing luteinisation. Small hilar cyst at bottom. × 75.

FIG. 4.—Virgin treated with BP for 49 weeks. Luteoma top right. The rest of the ovary is composed of germinal epithelium, merging corpora lutea and an atretic follicle. × 75.

FIG. 5.—Virgin treated with DMBA for 11 weeks. Small luteoma with dead ovum in centre. × 125.

FIG. 6.—Virgin treated with MC for 56 weeks. Granulosa cell tumour of pseudofollicular pattern adjacent to telangiectatic area (part of which is seen top left). Rest of ovary shows thecal luteinisation. × 110.

FIG. 7.—Untreated virgin 55 weeks old. Atretic follicles, dead ova, intact corpora lutea, dark staining cells and groups of lipochrome-containing phagocytes. × 120.

FIG. 8.—High power view of the same ovary as in Fig. 3 showing wrinkled dead ovum, and bands of spindle thecal cells cut longitudinally and transversely. × 140.

FIG. 9.—Untreated virgin 98 weeks old. Large hilar cyst on the right, atrophic remnant of the ovary to the left. Wrinkled germinal epithelium, dark staining cells, lipochrome-containing phagocytes and one dead ovum at bottom right. × 70.

FIG. 10.—Virgin treated with DMBA for 23 weeks. Large numbers of dead and 2 calcified ova, between intact corpora lutea. × 80.

FIG. 11.—Virgin treated with MC for 55 weeks. Hilar cyst at top lined by columnar cells with vacuolated cytoplasm. Bands of luteinising thecal cells cut longitudinally and transversely. × 170.

3

4

Biancifiori, Bonser and Caschera.

5

6

7

Biancifiori, Bonser and Caschera.

8

9

10

11

disappeared but lutein cell cords persist and pigment-containing phagocytes and dark staining cells are abundant. There is often theca cell proliferation, anovular buds and hilar cysts (Fig. 3, 8 and 9).

### *Non-tumourous Effects of Chemicals on C3Hb Ovaries*

It is convenient to consider the effects of DBA and BP together, 50 and 32 ovaries respectively having been examined microscopically. Two main differences when compared with untreated ovaries of approximately the same age are seen (Table V) : the presence of the small luteomas already described and the absence of hilar cysts. Apart from this the ovaries may be considered as having undergone normal age changes.

TABLE V.—*Ovarian Changes at Different Ages*

| Chemical | Weeks following commencement of treatment | Dead and calcified ova | Atretic follicles | Persistence of corpora lutea | Anovular buds | Dark staining cells | Pigment containing phagocytes | Hilar cysts | Thecal proliferation |
|---|---|---|---|---|---|---|---|---|---|
| BP | 32–50* | + | Few | + | 0 | 0 | 0 | 0 | 0 |
| DBA | 33–54* | + | + | + | 0 | 0 | 0 | 0 | 0 |
| MC | 42–85* | Few | 0 | 0 | Few to + | + | 0 to ++ | + | + |
| DMBA | 11–41* | + to few | Few | + to few | 0 | 0 to + | 0 to + | 0 | 0 to + |
| None | 50–60† | + | + | + | 0 | 0 | 0 | + | 0 |
| | 78–100† | + | Few | 0 | + | + | + | + | Little |

* Add 12 weeks to give actual age.

† Weeks of age.

With MC and DMBA, however, the non-tumourous changes are different and will be described separately.

All the mice treated with DMBA were dead at 42 weeks after the beginning of treatment. As the large tumours occupied the whole ovary and as a few non-tumourous ovaries were not sectioned, the following description is based on 50 ovaries examined from 11 weeks onwards. Even at 11 weeks, dead and calcified ova are present, the remaining follicles are atretic and some of the corpora lutea are beginning to merge, though intact old corpora lutea are still present (Fig. 10). Anovular buds were absent throughout. At 19 weeks, thecal proliferation and luteinisation appears for the first time and becomes progressively more prominent as time proceeds, taking the place of the corpora lutea. Dead ova and small atretic follicles may still persist. Only occasionally are dark staining cells present in any numbers and hilar cysts were only seen in 3 of 50 ovaries. Thus the new feature of thecal proliferation and luteinisation is seen in ovaries treated with DMBA, from which dark staining cells, anovular buds and hilar cysts are absent (Fig. 11).

In Fig. 1 it is seen that two granulosa tumours arose at 11 and 12 weeks respectively, i.e. before thecal proliferation was observed. One of these occupied the whole ovary and in the other ovary no thecal proliferation was present alongside the small tumour in the section examined. This occurrence is not held to be sufficient to discount the idea that thecal proliferation is an essential precursor of tumour genesis.

From the 19 mice treated with MC, 17 ovaries not wholly occupied by tumour were examined at 53–85 weeks following the initiation of treatment. Dead and calcified ova are occasionally seen but atretic follicles and corpora lutea are entirely absent, their place being taken by thecal proliferation or, later, by pigment-containing phagocytes and dark-staining cells. Simple hilar cysts occur in nearly all the ovaries. Anovular buds increase in number as time proceeds but are never very numerous.

### *Other Tumours*

*Stomach.*—Keratinising squamous papillomas or carcinomas occurred in all the groups. The highest incidence (26 per cent) was in MC-treated mice, but the tumours appeared late (42–85 weeks). Two-thirds were malignant. A similar incidence (24 per cent) occurred in DMBA-treated mice, but at an earlier age (11–40 weeks). In BP- and DBA-treated mice the incidence was 11 and 3 per cent respectively, the single tumour in a DBA mouse being a simple papilloma.

*Liver.*—Hepatomas occurred in two MC-treated mice at 56 and 63 weeks but none were observed in the other groups, where the animals died at an earlier age. In two control mice aged 98 and 99 weeks hepatomas were also present.

### *Pseudopregnancy*

Fifteen normal C3Hb virgins were kept 5 in a cage. Daily vaginal smears were made for 60 consecutive days and the oestrous cycles were recorded (Table VI). Of 150 cycles, 21 were characteristic of pseudopregnancy, an incidence of 14 per cent. Mühlbock and Boot (1960) had previously found 12 per cent of pseudopregnancies in 332 cycles in C3Hf mice.

TABLE VI.—*Incidence of Pseudopregnancy in C3Hb and BALB/c Mice*

| Strain | Number of mice examined | Number of cycles recorded | Number of pseudo-pregnancies | Per cent of pseudo-pregnancies |
|---|---|---|---|---|
| C3Hb . . . . | 15 | 150 | 21 | 14 |
| BALB/c (Caschera, 1960) . | 50 | 250 | 10 | 4 |

### DISCUSSION

The response of virgins of the C3Hb strain to treatment with chemical carcinogens must be discussed in relation to what is known about the strain itself and what is known about the response of other strains to chemical carcinogens.

### *Comparison of spontaneous and chemically induced mammary and ovarian tumours in C3Hb mice*

(a) *Mammary.*—No tumours occurred in the 20 control mice listed in Fig. 1 and 2 although 5 mice lived to the age of 98 weeks. Mammary tumours in Heston's (1958) virgin agent-free C3H mice occurred at an average age of 22 months and in Deringer's (1959) line at 20·7 months.

There is thus no doubt that the chemically induced tumours (Table II) occurred at an earlier period than the spontaneous ones. As the number of tumours induced with each chemical was so variable, ranging from 54·3 per cent following MC to

3·6 per cent following BP, it is concluded that these tumours were specifically induced by the chemicals. Morphologically the induced tumours had a greater variety of structure (Table III) than did spontaneous tumours. Only 20 of 51 tumours presented squamous metaplasia.

(b) *Ovarian.*—Among 20 untreated virgins (Fig. 1 and 2) a single microscopical granulosa cell tumour was observed at 76 weeks of age and 3 microscopical luteomas at 52, 56 and 86 weeks. Following chemical treatment the incidence of these types of tumour is greatly raised, the induction period is reduced and there is a dissociation of both these events according to the chemical used (Fig. 1 and 2). These facts lead to the conclusion that the chemicals specifically induce ovarian tumours. If the two types of tumour are considered separately it is clear that MC fails to induce luteomas whereas the other three chemicals are active in the order BP, DBA and DMBA. The latter chemical induces granulosa cell tumours early, whereas MC induces them late, and DBA and BP not at all. The size of the granulosa cell tumours is related to their yield (Table IV), the large tumours being found in mice treated with DMBA and MC.

*Comparison of chemically induced mammary and ovarian tumours in different strains*

(a) *Mammary.*—There is an interesting difference in the response of IF and C3Hb virgins to the four chemicals : in IF mice BP was the least potent chemical (15 per cent of tumours occurring after 36 weeks) whereas DMBA, MC and DBA each induced approximately 40 per cent of tumours. There was dissociation of induction time, DMBA and MC tumours occurring before 36 weeks and DBA tumours after (Bonser, 1958). In the present experiments, the range of tumour incidence was greater (MC 54, DMBA 31, DBA 14 and BP 4 per cent respectively). The DMBA tumours all occurred before 40 weeks but the majority of MC-induced tumours occurred after 40 weeks. It seems unlikely that the differing method of administration (painting in IF mice and oral in C3Hb mice) would account for this difference.

(b) *Ovarian.*—The important difference between IF and C3Hb mice in this respect is the high yield of granulosa cell tumours following MC treatment (9 large tumours in 19 mice). However, these tumours occurred after 50 weeks, whereas all the IF mice were dead at 48 weeks, and thus the time factor is probably of importance. The high early yield of tumours following DMBA treatment is comparable with that in IF mice.

Among the mice bearing granulosa cell tumours following treatment by MC two examples of large bilateral tumours were encountered, and one of a large tumour on one side and a microscopical tumour on the other. Large bilateral tumours were not encountered following DMBA treatment of IF mice (Marchant, 1959) or of C3Hb mice in this experiment.

(c) *Dissociation of mammary and ovarian tumours.*—Although both mammary and ovarian tumours might occur in the same mouse, as noted by Marchant (1959) and by Streeter (1960) in IF mice, there was no significant association between the occurrence of the two types of tumours.

(d) *Relation to pseudopregnancy.*—The incidence of pseudopregnancy in control virgin C3Hb females kept under the same conditions as the treated mice, is 14 per cent. As normal cycles last for about 5 days and each pseudopregnancy for about 12 days, the breast is under the influence of functioning corpora lutea for

25–30 per cent of the experimental period. In BALB/c mice only 4 per cent of the cycles are pseudopregnancies (Table VI) so that corpora lutea function only for about 12 per cent of the experimental period. Virgin BALB/c mice do not develop mammary carcinomas when treated with MC, in contrast with C3Hb and IF mice (44 per cent of pseudopregnancies (Mühlbock and Boot, 1960) which do. These findings support the work of Jull (1954) who found that oestrogen alone was not a sufficient hormonal stimulus to cause mammary tumours in castrated IF females when treated with a limited dose of the carcinogen MC but that the addition of progesterone resulted in an incidence of 82 per cent of mammary carcinomas.

*Genesis of the ovarian tumours.*—As normal C3Hb ovaries age they show two features not described by Mody (1959) in IF ovaries : calcification of the ova and hilar cysts, the latter probably derived from rete tubules. These structures are continuously present from 50–100 weeks, no information being available about the state of the ovary before this time. C3Hb ovaries age later but in the same manner as IF ovaries (Table V). When treated with chemicals the non-tumourous ovaries either age in normal fashion except for the lack of formation of hilar cysts (DBA and BP), or undergo a more rapid ageing process (MC and especially DMBA) associated with a proliferation of thecal cells. Mody thought that this was the essential precursor to the formation of tumour nodules but no direct evidence for this view has been derived from the present experiments.

Granulosa cell tumours were seen in all stages of development, from small nodules to large tumours. Occasionally luteinised areas were seen in these tumours and thus they might be called " mixed ". The luteomas were all either microscopical or small ; the largest measured 2·8 mm. in diameter (a diameter partly accounted for by the central haemorrhage) and the others 1·0–2·0 mm. As they occurred most frequently in DBA- and BP-treated mice, in which thecal proliferation is usually absent, it is unlikely that they arose in the same way as the granulosa cell tumours. From their early microscopical appearance and as the remnant of an ovum was found in the centre of two it seems possible that they arose in damaged follicles. No opinion can be expressed as to their autonomy.

### SUMMARY

Virgin milk-agent-free C3H mice, named C3Hb and originally obtained from Andervont, were given a limited dose by oral administration* of 4 chemical carcinogens : DMBA, MC, DBA and BP.

C3H virgins when kept 5 in a cage undergo 14 per cent of pseudopregnancies.

Three luteomas and one granulosa cell ovarian tumour (all microscopical in size) occurred in 20 control virgins living to a greater age than the treated mice (Fig. 1 and 2). No mammary tumours occurred. Mammary and ovarian tumours occurred in breeding females of other lines of the strain maintained in the U.S.A.

C3Hb ovaries age in the same way but at a later date than do IF ovaries, with the exception of two characters : calcification of the ova and the formation of hilar cysts (Table V).

The ovarian tumours induced by DMBA (68 per cent) and MC (46 per cent) were of granulosa cell type, the former occurring earlier than the latter. Those induced by BP and DBA were luteomas.

* Half the DBA- and BP-treated groups were painted.

The granulosa cell tumours arose in areas of thecal proliferation after the follicles and corpora lutea had disappeared. The luteomas probably arose in damaged follicles. No evidence was obtained that granulosa cell tumours became luteomatous or *vice versa.*

Mammary tumours were induced by all four chemicals in the following descending percentage: DMBA (52·9), MC (31·4), DBA (13·8) and BP (3·6). DMBA tumours occurred earlier than all the others. The susceptibility of the mammary gland to chemical carcinogens is related to the fact that virgin mice undergo spontaneous pseudopregnancy.

C. Biancifiori and F. Caschera were supported by Grant C–3844 ( (C1), National Cancer Institute, National Institutes of Health, Public Health Service, Bethesda, Maryland, U.S.A.

### REFERENCES

ANDERVONT, H. B. AND DUNN, THELMA B.—(1948) *J. nat. Cancer Inst.*, **8**, 227.—(1950) *Ibid.*, **10**, 895.—(1953) *Ibid.*, **14**, 329.

BIANCIFIORI, C., BONSER, G. M. AND CASCHERA, F.—(1959) *Brit. J. Cancer*, **13**, 662.

BIELSCHOWSKY, F.—(1958*a*) *Rep. Brit. Emp. Cancer Campgn*, **36**, 456.—(1958*b*) *Proc. int. Symp. on Mammary Cancer*. Ed. L. Severi. Perugia. p. 481.

BONSER, GEORGIANA M.—(1958) *Ibid.*, p. 575.

BOOT, L. M. AND MÜHLBOCK, O.—(1956) *Acta Un. int. Cancr.*, **12**, 569.

CASCHERA, F.—(1960) *Lav. Ist. Anat. Univ. Perugia*, **20**, 17.

COMMITTEE ON STANDARDIZED GENETIC NOMENCLATURE FOR MICE.—(1960) *Cancer Res.*, **20**, 145.

DERINGER, MARGARET K.—(1959) *J. nat. Cancer Inst.*, **22**, 995.

HESTON, W. E.—(1958) *Ann. N.Y. Acad. Sci.*, **71**, 931.

HOWELL, J. S., MARCHANT, J. AND ORR, J. W.—(1954) *Brit. J. Cancer*, **8**, 635.

JULL, J. W.—(1954) *J. Path. Bact.*, **68**, 547.

MARCHANT, JUNE.—(1957) *Brit. J. Cancer*, **11**, 452.—(1959) *Ibid.*, **13**, 652.

MODY, JER.—(1959) Thesis submitted to the University of Leeds for the degree of Ph.D. —(1960) *Brit. J. Cancer*, **14**, 256.

MÜHLBOCK, O. AND BOOT, L. M.—(1960) Symposium on ‘ Phenomena of the tumor viruses.’ New York City.

STREETER, DOROTHY J.—(1960) Thesis submitted to the University of Leeds for the degree of M.Sc.